# A Practical Guide to Rodent Islet Isolation and Assessment Revisited

**DOI:** 10.1186/s12575-021-00143-x

**Published:** 2021-03-01

**Authors:** Kathryn L. Corbin, Hannah L. West, Samantha Brodsky, Nicholas B. Whitticar, William J. Koch, Craig S. Nunemaker

**Affiliations:** 1 Heritage College of Osteopathic Medicine, Ohio University, Athens, OH USA; 2 Department of Biomedical Sciences, Ohio University, Athens, OH USA; 3 Honors Tutorial College, Ohio University, Athens, OH USA; 4 Translational Biomedical Sciences Program, Graduate College, Ohio University, Athens, OH USA; 5 Diabetes Institute, Ohio University, Athens, OH USA

**Keywords:** Islets of Langerhans, Isolation, Beta cell, Collagenase, Density gradient, Neonatal, Calcium, Insulin secretion

## Abstract

Insufficient insulin secretion is a key component of both type 1 and type 2 diabetes. Since insulin is released by the islets of Langerhans, obtaining viable and functional islets is critical for research and transplantation. The effective and efficient isolation of these small islands of endocrine cells from the sea of exocrine tissue that is the rest of the pancreas is not necessarily simple or quick. Choosing and administering the digestive enzyme, separation of the islets from acinar tissue, and culture of islets are all things that must be considered. The purpose of this review is to provide a history of the development of islet isolation procedures and to serve as a practical guide to rodent islet research for newcomers to islet biology. We discuss key elements of mouse islet isolation including choosing collagenase, the digestion process, purification of islets using a density gradient, and islet culture conditions. In addition, this paper reviews techniques for assessing islet viability and function such as visual assessment, glucose-stimulated insulin secretion and intracellular calcium measurements. A detailed protocol is provided that describes a common method our laboratory uses to obtain viable and functional mouse islets for in vitro study. This review thus provides a strong foundation for successful procurement and purification of high-quality mouse islets for research purposes.

**Supplementary Information:**

The online version contains supplementary material available at 10.1186/s12575-021-00143-x.

## Introduction

In early 1869, with his mention of “small cells of almost perfect homogeneous content, and of perfect polygonal form … lying together in twos or in small groups,” [1] Paul Langerhans quietly announced to the medical community the discovery of what would later become known as the “islets of Langerhans”. This announcement would also set in motion the groundwork for successfully treating diabetes mellitus [2, 3]. At the time of the discovery, the function of these small clusters of cells was unknown, and Langerhans did not speculate about it. Twenty years would elapse between Langerhan’s initial discovery and the connection between the pancreas (and by default, islets) and diabetes was clearly established by Oskar Minkowski and Josef von Mering in 1889 [4]. By 1893, Gustave Édouard Laguesse hypothesized that the islets of Langerhans produced “internal secretions” that Jean de Meyer named “insulin” in 1909 [5]. Attempts to isolate this “internal secretion” began as early as 1907 but were not successful until the 1920s when insulin was first used to treat diabetes in a human patient [2, 5].

 It was hypothesized that the exocrine tissue would impair the vitality and function of the endocrine pancreas [5, 6]. In 1902, Leonid W. Ssobolew suggested that the separation of the exocrine pancreas from the endocrine would increase islet viability and improve success in transplantation procedures [5, 6]. To this end, R. R. Bensley pioneered the staining of islets using neutral red and hand picking [5, 7]. But here the isolation process languished until 1964, when Claes Hellerström developed a technique using microscope microdissection [8]. In swift succession, beginning in 1965, isolation techniques evolved using collagenase [9], utilizing the anatomy of the pancreas in 1967 [10], and a density gradient in 1969 [11]. These three techniques were finally pulled together in 1985 by Gotoh et al [12] and truly opened the way for islet transplantation and in-depth study of islets.

**Fig. 1:**
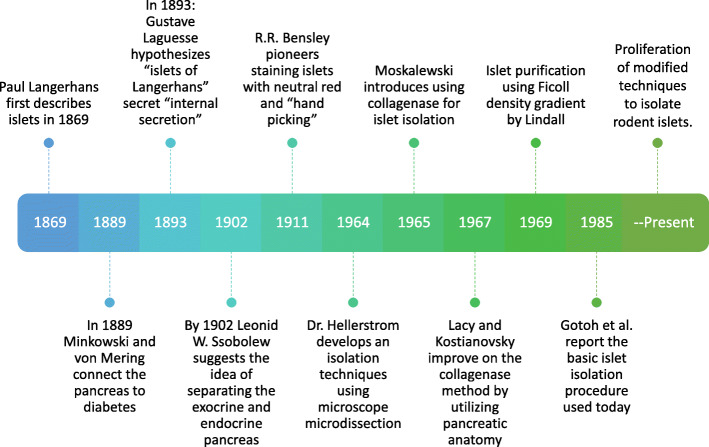
Timeline of advances in the understanding of islets of Langerhans

Figure 1 shows this flow of advances in islet isolation. Since that time, there have been no major changes in the basic approach to islet isolation procedures. Our lab provided “A Practical Guide to Rodent Islet Isolation”, describing our insights into these procedures in 2009 [13]. Other groups have looked at a variety of digestive enzymes [14–16], variations on delivery of digestive enzyme [14, 17], and the separation of islets from exocrine tissue [18–20]. Some researchers have advocated the use of filtration to purify islets, skipping the density gradient completely [21, 22]. There have also been efforts to use stem cells [23–25] or dissociated islet cells and human amniotic epithelial cells [26], thus attempting to reduce or eliminate the need for islet isolations.

Islet isolation was pioneered in a very narrow range of species, specifically guinea pigs [7, 9–11, 27, 28] and mice [8, 12], with mice and rats still being the most often used species [16, 29–31]. Pigs [25], goats [32], cats [33], monkeys [34, 35], and dogs [36, 37] are also used to study various aspects of diabetes and potential treatments.

**Fig. 2:**
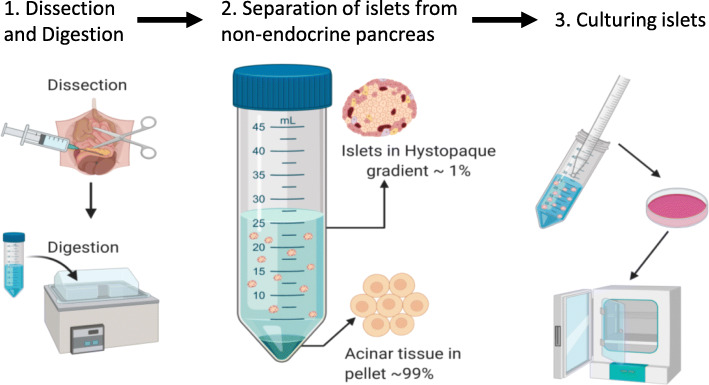
A graphical representation of the three key elements for a successful islet isolation. (1) The successful dissection of the pancreas followed by digestion. (2) Separation of the islets from acinar tissue using a separation gradient. It should be noted that islets are not visible to the naked eye and are thus not to scale in the illustration. (3) Culturing islets to ensure viability and functionality

The primary goal of islet isolation, whether for in vitro studies or for transplantation, is to obtain purified islets that are both viable and responsive to stimulation in a manner consistent with their function in vivo. To this end, we highlight three key elements of a successful islet isolation procedure: 1) Digesting the tissues connecting the islets to the exocrine tissue, 2) separating islets from non-islet tissue, and 3) culturing islets in an environment that maintains viability and function (Fig. 2). This paper will review these key elements and provide detailed methods used in our laboratory to consistently procure viable and functional islets for research. Our protocols are by no means the only successful methods for the isolation of pancreatic islets; however, the additional details provided in these protocols for each step will hopefully assist researchers in their efforts to obtain a large number of healthy islets for both study and transplantation.

## Procedures of Islet Isolation

### Basic Methodologies

When assessing and evaluating any islet isolation protocol consideration must be given to differences in the type and concentration of collagenase, the method of administration of the collagenase, temperature and duration of the digestion phase, the method used to purify islets from pancreatic acinar tissue, and the culture conditions following isolation. Each of these considerations are discussed below. While these items were discussed in our previous guide [13], each has been expanded, providing more up to date information for those who are new to the study of islets.

A survey of the literature for rodent islet isolation reveals a variety of methods for delivering the digestive enzymes to the pancreatic tissue surrounding the islets as well as different digestive enzymes. One method of delivery is to excise the pancreas from a euthanized animal and cut it into pieces, increasing the exposed surface area and providing improved conditions for the digestive enzyme to break down the tissue surrounding the islets [10, 17, 38]. The pieces are then enzymatically digested in the solution while also being mechanically digested by either shaking or stirring. A second method, described by Gotoh et al, has collagenase injected into the common bile duct of a euthanized animal. The inflated pancreas is then excised and digested at 37 °C without being cut into pieces or the addition of mechanical digestion [39].

While these two approaches form the foundation of many islet isolation techniques, there are considerable variations among published methods as well as alternative methods [8, 16, 20, 29, 40–43]. The advantages of using the common bile duct are twofold: 1) it allows the digestive enzyme to access the islets using the anatomical structures of the pancreas, and 2) stationary digestion reduces mechanical damage to the islets. Our laboratory uses a modification of the common bile duct protocol when isolating rodent islets using collagenase (see Appendix A for an annotated murine protocol and Appendix B for a bullet point protocol). Although cannulation of the murine bile duct requires technical skill, we are partial to this approach, as the collagenase interacts more closely with the connective tissue surrounding the islets when it is delivered through the intact pancreatic ductal system [44–46], resulting in a greater number of successfully isolated islets. There are several detailed online video accounts of a rodent islet isolation using a method of bile duct cannulation [16, 29, 30, 47–49].

### Collagenase – Selection and Use

Collagenases are proteolytic enzymes that digest collagen, a major structural protein in animals [50, 51] and have been found in everything from bacteria to mammals [50–53]. Out of all the potential sources, it was found that all collagens or collagen-like proteins are susceptible to the enzyme obtained from *Clostridium histolyticum* (*Cl. histolyticum*) [51], the bacterium from which collagenase was first purified in the 1950s [54]. Subsequent studies found the raw collagenase obtained from this bacterium is a mixture of several different collagenases distinguished by molecular mass, termed alpha, beta, gamma, delta, epsilon, zeta, and eta [55, 56]. These have been at least partially characterized for stability and specificity [54, 55, 57–61]. It was also found that these collagenases could be separated into two distinct classes based on substrate specificity and amino acid analysis [58]. Class I collagenase encompasses the isoforms designated alpha, beta, gamma, and eta [56]. These four have high collagenase activity and moderate activity on the synthetic peptide 2-furanacryloyl-L-leucylglycyl-L-prolyl-L-alanine (FALGPA) [62]. The class II collagenases are delta, epsilon, and zeta [56] and have moderate collagenase activity and high FALGPA activity [62]. After these classifications were made, it was found that *Cl. histolyticum* has two distinct, yet separate genes that code for collagenase. The gene designated *colG* codes for those collagenases in class I while the *colH* gene codes for class II collagenases [63]. Since it is difficult to completely purify one type of collagenase from another, and the enzymes act synergistically, characterization is of vital importance.

Since collagenase is purified from bacterial culture, considerable variability exists between manufacturers as well as between production lots of collagenase from the same manufacturer. Therefore, the formulation, enzyme activity and purity of a given lot all strongly influence the outcome of any islet isolation. The composition of the collagenase and the concentration of enzymes in a specific lot must therefore be ideally suited to the task of islet isolation. A detailed description of the differences in isolation with purified collagenase type I and II as well as the combination that yields the most effective isolation of rat islets have been described by Wolters et al [64, 65]. Others formulated criteria for evaluating each lot of commercially available collagenase to ensure proper digestion of rat islets [66]. Those collagenase formulations with increased collagenase activity, low levels of trypsin activity, and a specific range of both neutral proteases and clostripain may yield the most viable islets [64, 66]. Our lab has found that optimal collagenase formulations used for rat islet isolation also provide acceptable criteria for rating collagenase used in our mouse islet isolation procedure.

Thus, there is a remarkable range of digestive enzyme formulations available for islet isolation, from crude collagenases to highly purified combinations used extensively for human islet transplantation. It has been suggested that the differences from lot to lot may be due to the lack of proper measurement of trypsin-like activity, even in the highly purified mixtures used in the isolation of human islets for transplantation [67]. Brandhorst et al suggested the trypsin-like activity in enzyme blends may work in concert with the other enzymes to increase the activity of the digestion, but there is some debate about the damage the trypsin-like activity has on the islets [68]. To ensure consistent activity and reproducibility for human islet isolation, the enzyme blends with both high purity and a precise notation of components are used, such as collagenase NB1 (Nordmark, Uetersen, Germany), Liberase (Roche, Basel, Switzerland) and Vitacyte HA (Vitacyte, Indianapolis, IN, USA) [67, 69–71]. These higher purity, and consequently high priced, enzymes are also used in human islet isolation to reduce the incidence of contamination by endotoxins [71, 72].

It has been shown that the presence of endotoxins correlates with an increase of the proinflammatory cytokines interleukin-1beta (IL-1beta), interleukin-6 (IL-6), and tumor necrosis factor-alpha (TNF-alpha) in both rat and human islets [69, 73, 74]. Both collagenase formulations and the different types of gradient compounds have been found to contain endotoxin in varying amounts [69]. Endotoxins are of particular concern in human islet transplant procedures especially since the infiltration of inflammatory cytokines in transplanted islets has been linked to endotoxin contamination in both enzyme blends and separation gradients [19, 69, 73, 74].

### The Process of Collagenase Digestion

**Fig. 3:**
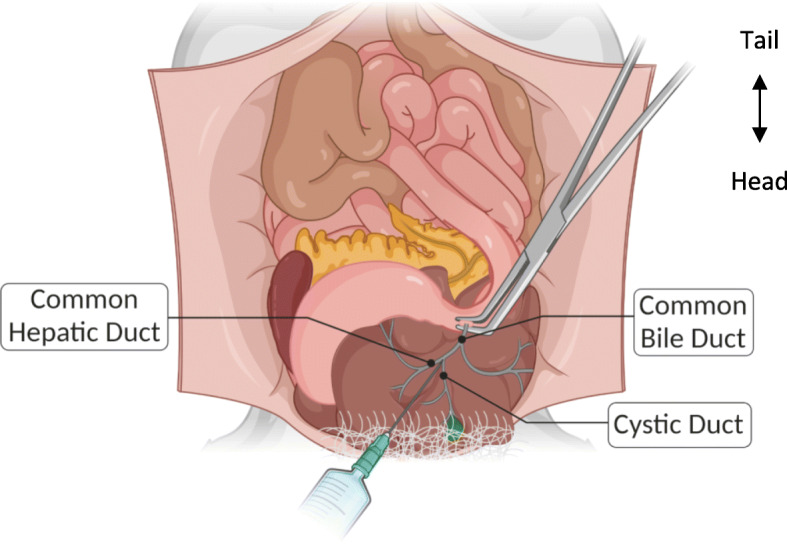
Anatomy of the mouse upper intraperitoneal cavity showing the injection site and clamping of the common bile duct at the duodenum. NOTE: The top is the caudal portion of the mouse, bottom is the rostral portion, i.e., this is from the perspective of the mouse lying on its back tail away from the surgeon and nose toward the surgeon.

The process of collagenase digestion is of the utmost importance to the success of any given protocol. To ensure success, consideration must be given to the type of collagenase, its activity, the concentration of the collagenase solution, its route of administration, and the duration and temperature of the digestion. These factors can all vary widely among the various protocols. Clamping or tying off the ampulla of Vater where the hepatopancreatic duct connects to the duodenum (at the sphincter of Oddi) and perfusing the pancreas through the common bile duct allows collagenase to access the islets using the biological structures of the pancreas (Fig. 3). This method may change the duration of the digestion when compared to other methods. Differences in composition between lots of collagenase and other factors influence digestion as well. Thus, it is important to test each protocol for optimal viability and islet function, both of which remain paramount to the success and reproducibility of islet isolation.

**Fig. 4:**
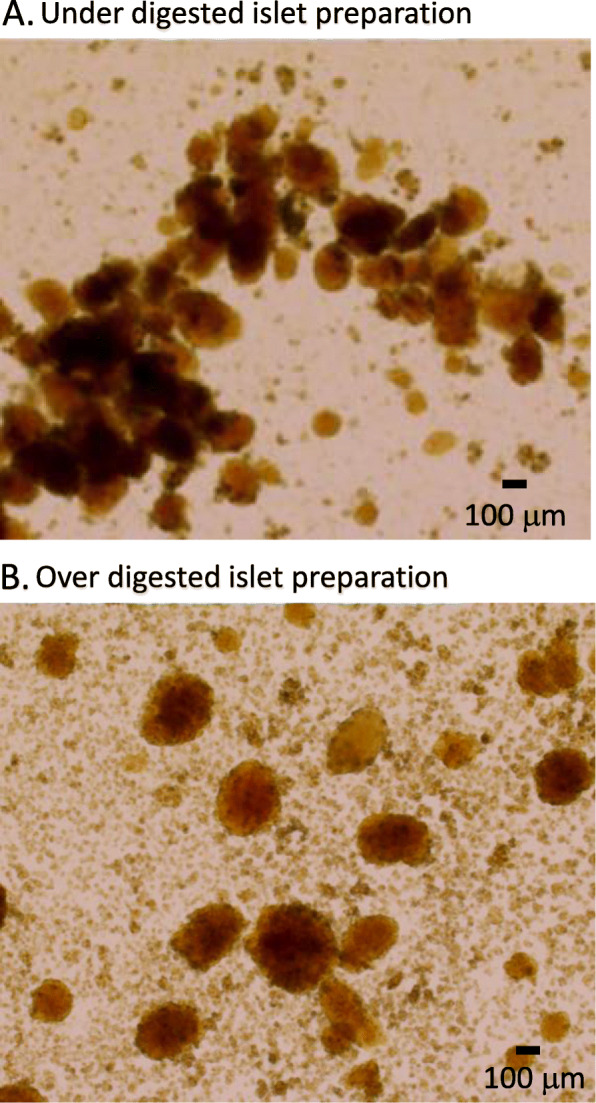
Examples of both under digested and over digested islets immediately post isolation and purification. **a** Under digested islets will typically contain large amounts of acinar tissue appearing dark brown or black. Islets are often aggregated near the masses of acinar tissue as shown. Separating islets from these under digested clumps is often difficult. **b** Over digested islet preparations result in large amounts of pancreatic debris consisting of tiny fragments of acinar tissue and digested islets

The duration of collagenase exposure is particularly important and should be optimized for each production lot. Figure 4 shows islets immediately post-separation when digestion time is not optimized to the unique collagenase lot. Digestion temperature, collagenase concentration, and route of administration were all the same; only digestion time was altered. In Fig. 4a, digestion time was not sufficient to separate the islets from the acinar tissue, resulting in clumping from which the islets will be difficult if not impossible to remove. These grossly encumbered islets will not be suitable for experiments. Figure 4b shows heavily over digested islets. Islets that have been over digested are fewer in number and typically either very large or very small. There also tends to be much finer debris in the plate as the islets will shed dead and dying cells for some time. In our experience, over digested islets are not as responsive to stimulation and do not survive overnight culture well.

Our lab, following published guidelines for collagenase enzyme formulations [66], uses Collagenase P (Roche, Basal, Switzerland, #11249002001) enzyme at 1.4 mg/mL in a modified Hank’s Balanced Salt Solution (HBSS) (Invitrogen, Carlsbad, CA, USA, #14065–056), injected into the pancreas via the common bile duct. The pancreas is then excised whole and placed in modified HBSS for stationary digestion at 37 °C for seven to 10 min as described in detail in Appendix A. Ideally, three separate digestions using two mice per time point should be completed to evaluate a newly purchased lot of collagenase. Our lab uses timepoints of 7, 8, and 9 min. After overnight incubation, islets from each digestion should be visually inspected and tested for viability and functionality using the assays reviewed below. The group with the highest yield (assuming proper cannulation) and best outcome in the assays indicates the proper digestion time.

### Separation of Islets and Pancreatic Acinar Tissue Using a Density Gradient

Purifying islets from acinar tissue, regardless of the method used, is important due to the nature of pancreatic tissue. The cells of the exocrine pancreas secrete various digestive enzymes that negatively impact islet viability [75]. Our lab has used both Ficoll 400 (MilliporeSigma, Burlington, MA) and Histopaque (MilliporeSigma, Burlington, MA) at different densities. In isolations using Ficoll 400 layered in a discontinuous gradient of 1.109, 1.096, 1.070, and 0.570 g/ml, islets were recovered from the interfaces of both the 1.070/1.096 and 1.096/1.109 g/ml layers. We found, however, that the preparations were often contaminated with acinar tissue. Using aseptically filled and premixed Histopaque, a mixture of sodium diatrizoate and Ficoll 400, we found that our purity results generally improved. Combining Histopaque 1.119 g/mL with the 1.077 g/mL preparation to produce a 1.100 g/mL gradient appears to enhance islet purity. It should be noted that our studies comparing Histopaque and Ficoll were not rigorous, and both gradients are widely used and accepted. McCall et al compared Histopaque, Ficoll, Dextran and Iodixanol assessing recovery, viability, purity, and in vitro functionality [76].

Depending on how much residual acinar tissue remains following the initial purification of the islets, a second purification is often needed to further increase the purity prior to culture. Our protocol includes using a basic light microscope with a 4x objective to identify islets, then handpicking those islets from one suspension culture dish into a second culture dish. Ideally, this is done 2 h post isolation. The islets then recover overnight in a 37 °C incubator with 5% CO_2_. The following morning, the islets are again handpicked into a new dish, thus eliminating any acinar tissue that may have either been transferred with the initial cleaning or was attached to the islets after isolation. This also removes healthy islets from any buildup of toxins from dying acinar tissue and from any necrosing islets that did not survive. It should be stated that once the islets are transferred to media, minimizing time outside the temperature-controlled and sterile conditions of the incubator will limit contamination and pH changes while handpicking islets for experiments.

### Islet Yields

Overall yield from a rodent pancreas is highly variable among strains. A comparison of seven different mouse strains was made by Bock et al and the number of islets per pancreas was found to range from ~ 1000 in 129S6 mice to ~ 2500 in B6 mice [77]. Using a mouse model of diabetes, they identified a similar number of islets per pancreas (~ 3200) for both the ob/ob and the ob/+ controls [78]. More recently, Mitok et al reported the average islet yield for eight different strains of mice, with > 400 islets/mouse from five of the strains (B6, A/J, WSB, CAST, and PWK), < 300 islets/mouse for the 129 and NOD strains, and notable sex differences in islet yield for the NZO strain [31]. De Haan et al investigated yields from different rat strains as well as factors that potentially influenced yields [66]. Inuwa et al demonstrated that the total number of islets in young male Wistar rats increased with age, ranging from ~ 6000 to ~ 20,000 islets per pancreas [79], while others have estimated the number per rat pancreas as low as ~ 3000–5000 islets [80, 81]. Thus, the expected islet yield from an isolation procedure depends a great deal on the age, sex, and strain of the rodent.

The expertise of the technician, as well as the method of isolation chosen, will also influence the total islet yield, making a definitive expected yield difficult to quantify. In our lab an experienced technician using 8–12-week-old CD-1 mice (Envigo, Indianapolis, IN) will usually obtain between 300 and 450 islets on average, while a similarly aged C57BL/6 J (Jackson Laboratory, Bar Harbor, ME) will yield only ~ 250–350 islets with an average yield of 300 islets per mouse. De Groot et al report rat yields of approximately 600–800 islets per animal [82]. Since the endocrine pancreas accounts for between 1 and 4% of the total volume of the pancreas [83], this suggests islet yields range from 10 to 30% from a typical rodent pancreas. For comparison, it is thought the human pancreas contains over one million islets, yields of approximately 250,000–300,000 islets are estimated by islet equivalents (IEQ) [84], although there are notable issues with IEQ quantification and no clear consensus on islet yields from human donors [85].

#### Conditions for Islet Culture

While our previous guide [13] contained information on conditions for islet culture, this guide has expanded that information as well as provided visual representations for islets at three different time points post-isolation. After an islet isolation, it is vital to ensure proper culture conditions so islets may recover from the process of collagenase digestion. An examination of media with different glucose concentrations showed that rat and mouse islets cultured with 11 mM glucose have lower apoptosis rates, increased viability, and increased immunoreactive insulin compared to islets cultured in either higher or lower glucose concentrations [86, 87]. Culture media with glucose concentrations substantially below 11 mM can reduce the insulin content in the islet as well as downregulate key genes related to glucose metabolism, whereas prolonged exposure to high glucose may lead to toxicity or other functional impacts [87–89]. Of all the culture media tested, RPMI 1640 supplemented with serum best maintained or augmented glucose-stimulated insulin secretion in murine islets [86]. Other groups have reported success in maintaining viability and function with rat islets in CMRL 1066 [66] and Iscove’s MEM [90].

Our lab uses RPMI 1640 (Invitrogen, Carlsbad, CA, USA #11875–093) culture media supplemented with 10% (v/v) fetal bovine serum (R&D Systems, Minneapolis, MN, USA #S11150) to promote viability and 100 U/mL penicillin and 100 mg/mL streptomycin (Invitrogen, Carlsbad, CA, USA #15140–122) to reduce contamination both for culturing of islets and while purifying islets from acinar tissue after density gradient separation. Islets are plated in 100 mm × 20 mm suspension culture dishes (Corning Inc., Corning, NY, #430591) as recommended [86], with 11 mL RPMI 1640 complete media in each dish.

**Fig. 5:**
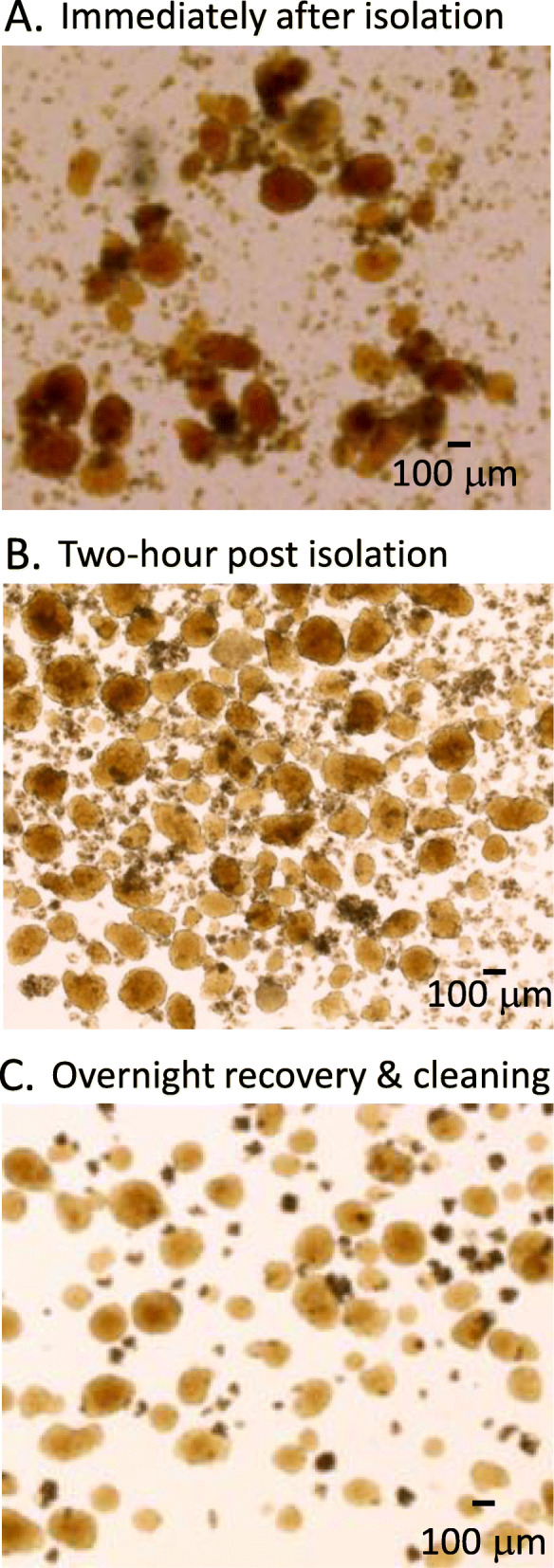
Islets after optimal digestion and recovery time. **a** An example of islets immediately post-isolation and purification. **b**-**c** The same preparation of islets after a two-hour recovery period but before cleaning (**b**) and after overnight recovery and before a final cleaning (**c**). Note that 88 islets were counted in (**b**) and 80 islets in (**c**) even though the tissue density appears quite different between images. Pancreatic debris accounts for the additional tissue observed in (**b**) prior to final cleaning

An optimal density of four islets per square centimeter has been suggested to prevent competition for nutrients [66]. Using the standard 100 mm × 20 mm culture dish with ~55cm^2^, this allows for ~ 220 islets per culture dish. Our lab prepares three culture plates per mouse, one for the islets immediately post separation, one plate for the initial hand cleaning of the islets two to 4 h post separation, and the last plate for the final cleaning after overnight (16–20 h) recovery. The initial 2–4-h recovery period allows for the islets to rest from the insult of collagenase digestion and to begin recovery. Cleaning the islets from the acinar tissue and any dead or dying islets increases the chance of islet survival. The second cleaning the following morning should result in a pure islet culture with little or no acinar tissue. Each of these time points are shown in Fig. 5. Islets immediately post separation (Fig. 5a) have some acinar tissue still evident on the islets. The same islets are shown in Fig. 5b after a two-hour recovery period and initial hand cleaning. Figure 5c shows the same islets after overnight recovery and hand picking a final time. Note there is still some acinar tissue present in the final image.

To maintain optimal islet health and function, storage in a sterile incubator at 37 °C with 5% CO_2_ and a humidified atmosphere is necessary [86]. Rodent islets can maintain glucose sensitivity for at least 1 week in culture with frequent media changes [86] and perhaps even longer based on data from human islets [86, 91]. It should be noted, however, that changes in rodent islet function can occur in as little as one to 4 days in culture [92].

##### Dispersion of Islets into Islet Cells

Although the intact islet provides an excellent model system for studying the function of this pancreatic micro-organ, some experimental approaches require dispersion of islets into individual islet cell cultures. Examining subtypes of islet cells requires islet dispersion as a requisite step. Thus, islet dispersion allows for the separation and examination of islet subpopulations such as alpha, beta, delta, and other islet cell types. Further, dispersion provides a convenient model of the isolated beta cell by preventing gap junction coupling [93, 94], blocking calcium waves between cells [95], and reducing paracrine signaling [96, 97]. This allows for comparisons between intact islets and dispersed beta cells [98]. A detailed protocol for dispersing, culturing, and identifying islet cells in culture is described in in Scarl & Koch et al. [99].

## Assessing Islet Health and Function

Once islets have been isolated and placed in culture, their health and function need to be assessed. In this section, we have updated and expanded the original description of assessment techniques presented in Carter et al [13].

### Morphology

**Fig. 6:**
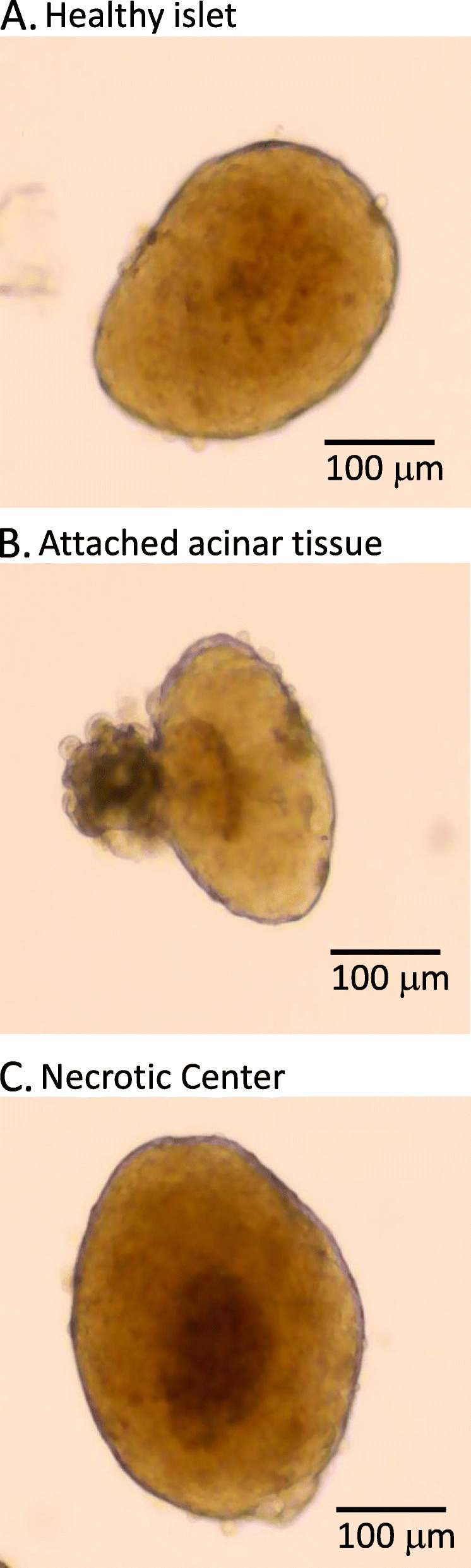
Islets found in a typical preparation. **a** Example of a healthy islet with amber color. **b** Example of an islet with acinar tissue, which typically appears as dark brown or black tissue on the exterior of the islet. **c** Example of an islet with a necrotic core due to hypoxia

Rudimentary information regarding the health of an islet may be provided by visual inspection. When viewed under a light microscope with 4x magnification, healthy rodent islets appear spherical and golden-brown in color, with an approximate diameter of 50–250 μm. These features, particularly the color when compared to the exocrine tissue, allow for rapid identification of islets. Healthy isolated islets following overnight recovery have few, if any, individual cells protruding from their relatively smooth surface, such as the islet in Fig. 6a. Cells protruding from the surface of an islet (Fig. 6b) can be a sign of decreased or decreasing health, as is acinar tissue clinging to the exterior of an islet (Fig. 6b). Larger islets are also prone to developing hypoxic cells in their core, visibly distinguished as a darker region when compared to the surrounding area (Fig. 6c). It has been suggested that hypoxia can be reduced by culturing islets at a lower incubation temperature initially [100] or by reducing the volume of media in the culture dish to increase oxygenation [101].

### Viability

To provide more quantitative measures of islet health than visual inspection alone, several techniques can be used to assess islet viability. We define viability as living versus dead or dying cells as assessed using cell exclusion or DNA-binding dyes. A common approach that is widely used in the human islet transplant field is to estimate the ratio of healthy living cells to dead cells within each islet by fluorescence microscopy. Fluorescein diacetate (FDA) incorporates into healthy cells by facilitated diffusion and fluoresces blue, and propidium iodide (PI) is a membrane impermeant red fluorescent dye that is excluded from viable cells and enters only dead or dying cells [102]. Using these fluorescent dyes in combination, the health of islets can be assessed by the FDA/PI ratio. Unmanipulated islets isolated from healthy control animals generally have 90–95% viability, meaning blue FDA staining in 90–95% of the component cells of an islet and red PI staining in only 5–10% of the cells in an islet. Additional techniques include AnnexinV (AnnV), SYTO-13/ethidium bromide, calcein AM/ethidium homodimer, fluorescein diacetate, and ethidium bromide [102–104].

**Fig. 7:**
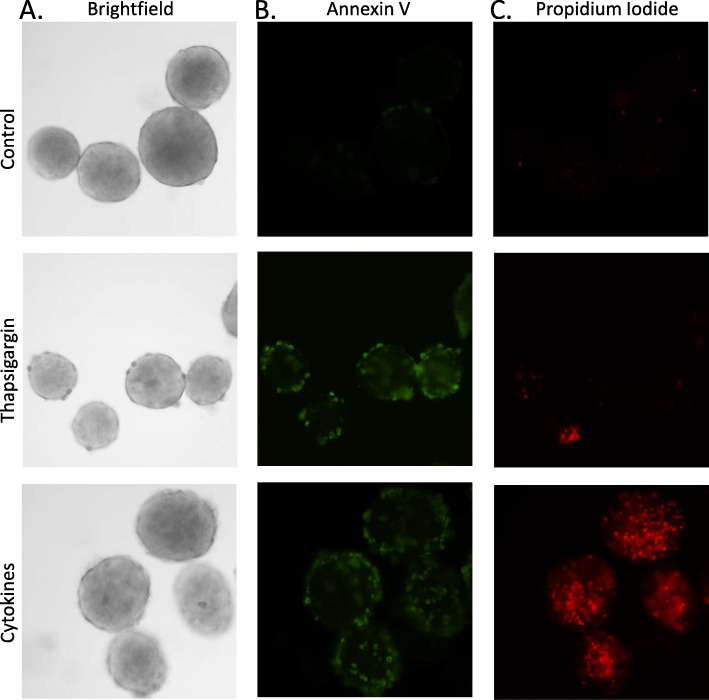
An example of thapsigargin-induced and cytokine-induced cell death as measured by annexin V and propidium iodide fluorescence. **a** A brightfield image of healthy mouse islets (top), islets treated overnight with 100 nM thapsigargin to induce ER stress (middle), and islets treated overnight with 5 ng/mL IL-1beta and 10 ng/mL TNF-alpha (cytokines) to induce cell death (bottom). **b** Annexin V is depicted with green fluorescence and propidium iodide is depicted with red fluorescence (**c**)

Rodent islets in a good preparation are uniformly viable at > 95%, so we primarily focus on quantifying cell death only. We use a variation of the above approach by quantifying the mean intensity of AnnV fluorescence to measure apoptosis and PI fluorescence to measure generalized cell death within an islet (see Appendix C). A region of interest is drawn around the perimeter of each islet using imaging software (CellSens, Olympus, USA) to quantify the fluorescent intensity of cell death signal per unit of cross-sectional area for each islet. Figure 7 provides an example of typical staining patterns for cell death following overnight culture in normal conditions (6A, top), ER-stress inducer thapsigargin (6A, middle), and proinflammatory cytokines (6A, bottom). As shown in Fig. 7b, almost no AnnV staining was observed in controls (top), whereas substantial AnnV staining was observed in the other conditions. In Fig. 7c, virtually no fluorescence is detected in control islets (top), and only one region on one islet from the thapsigargin-treated group showed any PI staining (middle). In contrast, PI staining was extensive among cytokine-treated islets (Fig. 7c, bottom). These observations are consistent with established apoptosis-specific pathways of thapsigargin-induced cell death [105] versus apoptotic and necrotic pathways activated by cytokines [106, 107]. We have published this approach previously to measure islet cell death in response to proinflammatory cytokines [108, 109] and high concentrations of metformin [110]. It should be noted that isolated islets, having lost vascular connection, can become severely hypoxic. This results in necrosis, especially in the core of the islet. Identification and removal of these islets from experiments can help in acquiring more accurate experimental data (see Supplemental Fig. S1 for additional details).

### Glucose-Stimulated Insulin Secretion (GSIS)

Insulin is crucial in regulating blood glucose, and reduced insulin secretion is a key feature of both type 1 and type 2 diabetes [111]. A fundamental property of islets is their capacity to regulate insulin release in direct response to changes in extracellular glucose concentrations. This ability defines islet function since insulin is produced and released only from islet beta-cells. There are other peptides produced by islet cells, such as glucagon, somatostatin, ghrelin, pancreatic polypeptide, as well as numerous “nonclassical” peptides [112]. These peptides are secreted in smaller amounts than insulin and are potentially more difficult to detect. Glucose-stimulated insulin secretion (GSIS) is thus a well-accepted measure of islet function and is considered the gold standard.

GSIS may be measured either by static conditions or by perfusing islets to measure the kinetics of insulin release in response to glucose. Each technique has its advantages and disadvantages which are reviewed elsewhere [113]. Typically, to measure functional GSIS, islets are cultured in a ‘low’ glucose concentration near 3 mM. This is to measure the amount of insulin secreted into the supernatant under ‘basal’ or ‘unstimulated’ conditions. Stimulated insulin release is then measured by exposing the islets to a higher glucose concentration such as 11 mM (half-maximal) or > 20 mM (maximal). In response to glucose stimulation, the time course of the islet response is biphasic, with a rapid spike in insulin (first phase) followed by a decline to a prolonged plateau (second phase) that remains for the duration of the stimulus. These are used to obtain a stimulation index (SI), the ratio of stimulated-to-basal insulin secretion. Healthy islets have an SI of 2–20 depending on several factors including strain, age, body weight, and glucose concentrations selected [82, 90].

One of the drawbacks to GSIS is the large number of islets required to conduct a thorough assessment. In recent years, the development of micro-plate assays using one islet per well have been used in an attempt to reduce the number of islets required for assessment, especially with regard to human islet transplantation [114]. Another point of contention is how best to control for variations in insulin secretion amongst islets to present the data obtained from a GSIS assay. In Slepchenko et al, we compared several normalization processes including total protein, insulin content, and islet area [115]. We concluded that normalization is not helpful in most cases as long as islets are within a size range of approximately +/− 50% of the mean because insulin secretion appears to be generally independent of islet size [115]. Among the normalization methods available, measuring the cross-sectional islet area was optimal for normalizing insulin secretion [115].

### Intracellular Calcium Imaging

Another important method to assess islet function is measuring changes in intracellular calcium ([Ca^2+^]_i_) in response to glucose or other stimuli [116]. We have used the ratiometric probe Fura-2 AM with an epifluorescent microscope [98], but there are many other calcium probes with different excitation and emission spectra that are capable of detecting changes in [Ca^2+^]_i_ including fluo-3, fluo-4, and fura red. Improved versions of some of these fluorophores have reduced leakage and photobleaching [117, 118]. Transfectable probes that target specific calcium-containing organelles such as the endoplasmic reticulum and mitochondria are also available [119, 120]. These more sophisticated probes require Forrester resonance energy transfer (FRET).

**Fig. 8:**
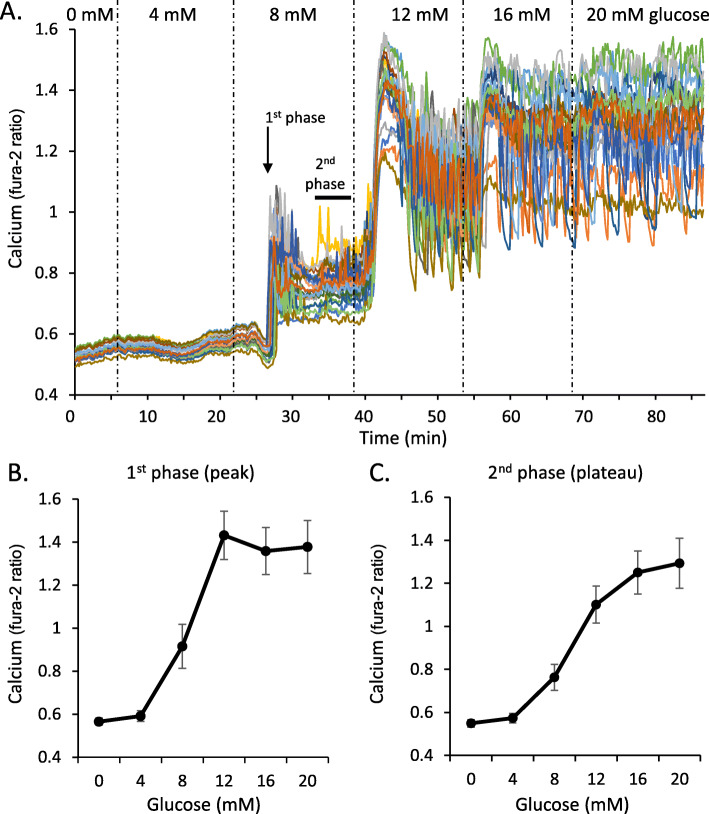
Calcium response to glucose stimulation. **a** Calcium traces recorded at 10-s intervals during a series of increasing glucose steps (0, 4, 8, 12, 16, 20 mM) in an 90-min recording (*N*=20 islets). For each glucose step both 1st phase (peak) and 2nd phase (plateau, measured in the last 5-min) were measured. **b**-**c** Mean values for each phase at each glucose step are used to assemble glucose dose-response curves for 1st phase (**b**) and 2nd phase (**c**) calcium responses as an indicator of islet function

We and others have found that Fura-2 AM can provide a basic picture of islet function and dysfunction [121–123]. As shown in Fig. 8a, changes in [Ca^2+^]_i_ can be assessed across multiple glucose steps to provide a detailed view of dose-dependent glucose stimulation. The 0 and 4 mM glucose concentrations are subthreshold, so there is little change in [Ca^2+^]_i_ activity as measured by the ratio of fluorescence intensity from 340/380 nm excitation light. The step to 8 mM glucose produces a classic biphasic response: an initial first phase peak followed by a second phase plateau (Fig. 8a). The 12 mM step provides the maximum first phase response in this example set of islets. The [Ca^2+^]_i_ levels continue to rise with each glucose step to maximum stimulation in 20 mM glucose. This ‘one-shot’ experiment provides enough data to produce a glucose dose-response curve for both 1st phase (Fig. 8b) and 2nd phase (Fig. 8c) [Ca^2+^]_i_. The procedures we used for [Ca^2+^]_i_ imaging can be found in Scarl & Koch et al. [99].

### The Use of Fluorescent Labels in [Ca^2+^]_i_ Experiments

**Fig. 9:**
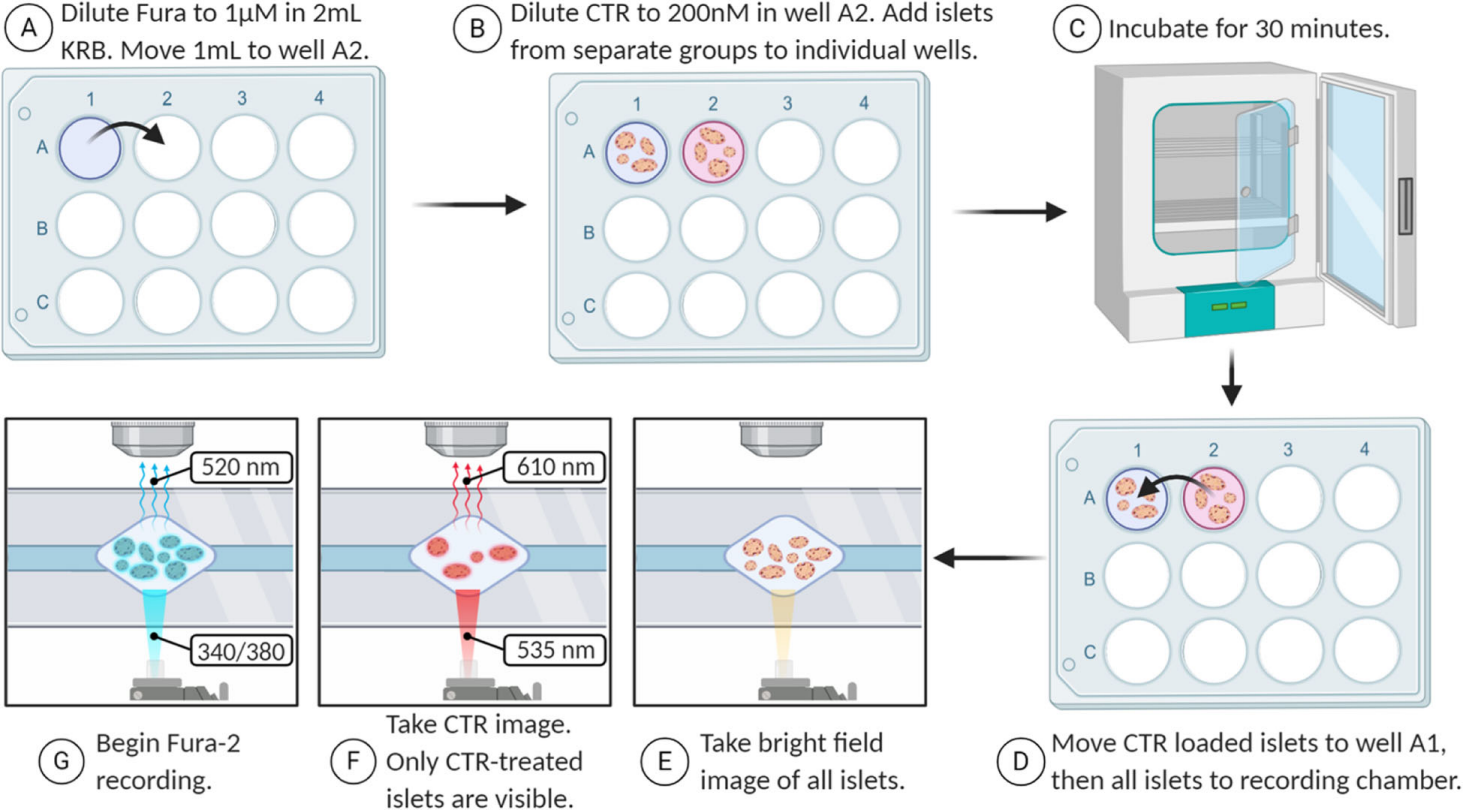
Fluorescence imaging with Fura-2 and CTR. Fura-2 is diluted in one well (**a**), then a 1 ml aliquot is moved to an adjacent well and CTR is added (**b**). Islets are added to each well and incubated for 30 min (**c**) before being combined in the Fura-2 only well and transferred to the recording chamber (**d**). Islets are imaged using three different wavelengths: bright field (**e**), CTR only (**f**), and Fura-2 (**g**). Once the calcium imaging experiment is complete, the islets from the second well can be identified as the red islets in the CTR image

To extend the value of [Ca^2+^]_i_ imaging as an evaluation tool of islet function, we utilize Cell Tracker Red (CTR) as a means to record and compare test groups side-by-side with control groups of islets. The basic approach was first described by Corbin et al. [124] and is shown in cartoon form in Fig. 9. Briefly, Fura-2 is diluted in one well of a 12-well plate (A), half of the solution is moved to an adjacent well, and CTR is added to the second well (B). Untreated or control islets are added to the first well while the ‘test islets’ (e.g. islets from a mutant mouse, islets treated with drugs to stimulate or inhibit insulin secretion, islets put under stress, etc.) are added to the second well and incubated for 30 min (C). At the end of the 30-min incubation (dye loading period) the islets are briefly combined in the Fura-2 only well (D) and then transferred to the recording chamber of the microscope. It should be noted that this will introduce a very small amount of CTR into the Fura-2 only well. This concentration is both extremely small and transient for the Fura-2 only islets since the fluid flow of the chamber will wash excess CTR and Fura-2 away. Islets are then located and imaged using brightfield illumination (E), then the red fluorescence is imaged from the CTR-labeled test cells only (F), and the [Ca^2+^]_i_ signal from all islets is imaged (G). Data sets can be separated based on the red fluorescence image, with islets that are not visible belonging to the well 1 control and islets with red fluorescence belonging to the well 2 test condition.

**Fig. 10:**
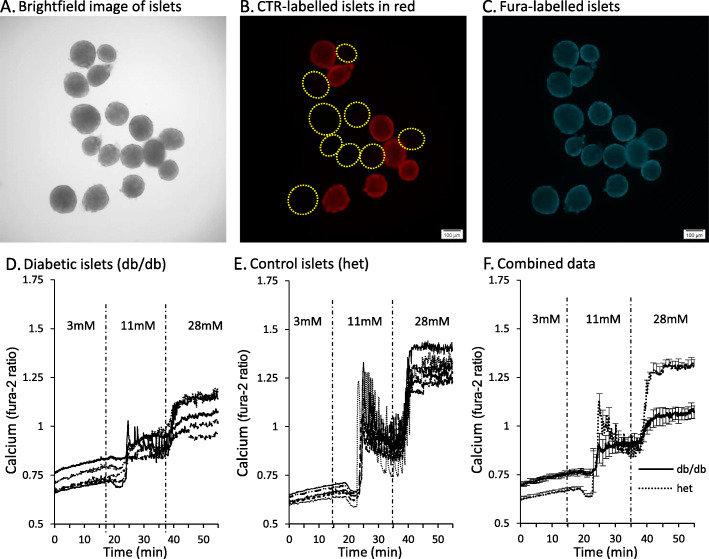
Example of Cell Tracker Red (CTR) being used to identify two different treatment groups of islets. **a**-**c** Images of islets: group 1 contains control islets loaded with Fura-2 for 30 min, and group 2 contains “test” islets loaded with Fura-2 and 200 nM CTR for 30 min. Both groups of islets are combined in the recording chamber for live-cell fluorescence microscopy. **a** A brightfield image shows all islets (group 1 and group 2). **b** An image taken with filters to detect only red fluorescence light (535 nm Ex; 610 nm Em). Test islets (group 2) produce detectable red fluorescence due to the CTR label. Yellow circles surround the location of “invisible islets” from group 1 that were not labeled with CTR. **c** Fluorescence from the Fura-2 signal (380 nm Ex) is detectable in all islets, so that calcium recordings of group 1 and group 2 can be made simultaneously under identical experimental conditions. **d**-**f** An example of a calcium recording using CTR to compare diabetic and healthy islets. **d** Calcium traces from diabetic islets isolated from db/db mice loaded with 1uM Fura-2 + 200 nM CTR for 30 min. **e** Calcium traces from healthy islets isolated from heterozygous control mice loaded with 1uM Fura-2 only. **f** Mean calcium traces for db/db (*N*=5 islets) and heterozygous controls (N=5) can be used to assess differences in amplitude, slope, and latency by directly comparing two treatment groups under identical environmental conditions

This CTR-labeling approach is displayed in Fig. 10. Here we show brightfield (A), red fluorescence (B), and Fura-2 signal (C) for sets of islets labelled with or without CTR. In Fig. 10d, we show [Ca^2+^]_i_ traces from a set of islets that were isolated from a diabetic db/db mouse and loaded with CTR and Fura-2. In Fig. 10e, we show [Ca^2+^]_i_ traces from the heterozygous controls that were loaded with Fura-2 only. As shown in Fig. 10f, the average responses were quite different, but the x- and y-axes accurately reflect the signal from both sets of islets. Recording control and test groups simultaneously controls for temperature, perifusion rate, and other variables, which allows us to identify subtle changes in islet function that are important when making statistical comparisons. Previous studies using this approach have identified signs of endoplasmic reticulum stress [109] and dissociations between insulin secretion and [Ca^2+^]_i_ [125] by examining the latency, amplitude, and slope of islet [Ca^2+^]_i_ responses to glucose stimulation. Although [Ca^2+^]_i_ can be a convenient and insightful indicator of islet health and function, it is important to also measure insulin secretion due to many facets of the insulin secretory pathway that are separate from changes in [Ca^2+^]_i_ [125–128].

## Conclusions

As stated previously, islet isolation is an intricate process, but the ability to consistently procure viable and functional islets is crucial to effectively study the physiology and pathophysiology of islets. In this review, we have addressed the key factors to consider in both the isolation and assessment processes to obtain healthy islets. We provide a method of isolation developed by integrating the reports of many others in the research field with careful experimentation to optimize the islet isolation process for our laboratory. While this protocol provides a start for islet isolation, any procedure must be optimized to the capabilities of the laboratory and the specific goals of the study.

## Supplementary Information

**Additional file 1: Supplemental Fig. S1** Hypoxic cell death as an unwanted variable in PI/AnnV studies. Due to loss of vascular connection during isolation, islets can become severely hypoxic, resulting in necrosis, especially in the core of an islet. Identifying and removing such islets from experiments helps in acquiring accurate data. (A) Brightfield image of untreated (control) islets (top) and cytokine-treated islets (bottom; overnight treatment with 5 ng/mL IL-1b and 10 ng/mL TNF-a). The smaller islets in both images have no evidence of hypoxia while the larger islets have clearly defined dark centers. (B) All cytokine treated islets show a distinct ring of AnnV staining indicative of cytokine-induced apoptosis on the outermost layer of cells. Note the dark centers do not stain for AnnV. (C) PI staining showing both cytokine -associated and hypoxia-associated cell death. The only islet in the control showing PI staining is the large, dark centered islet while the cytokine treated islets show both cytokine-induced and hypoxia-associated cell death. The small cytokine treated islets on the left show several distinct punctate PI- stained nuclei typical of cytokine effects. The larger islets show varying degrees of punctate surface staining indicative of cytokine-induced cell death, but a large central mass of PI staining corresponding to the dark centers observed in the brightfield image.

## Data Availability

Not Applicable. This is a review article.

## Appendix A: An Annotated Protocol for the Isolation of Islets from Mice

### Materials

1 Forceps.

1 Fine dissecting forceps.

1 Fine Iris scissors for internal use.

1 standard pattern scissors.

1–2 Bulldog clamp(s).

30 or 27G ½" needle.

5 mL Luer-lock syringe.

25 mL pipette.

10 mL pipette (1 per genotype/strain/sex/condition).

5 mL pipette.

15 mL conical tube (1 per mouse).

50 mL conical tube (2 per mouse).

2"× 2" gauze.

37 °C water bath.

Scale (accurately measures in mg).

Centrifuge.

**Solutions.**

For Anesthesia:

CO2 gas.

Or:

Solution (A): Mix 2 mL of ketamine and 1 mL of xylazine.

*The concentration of xylazine used is 20 mg/mL.*

Solution (B): Mix (A) and 7 mL of Normal Saline, inj.

*Various methods of anesthesia are used to ensure that research protocols follow humane methods. The following are used by our laboratory: CO*_*2*_ *gas for euthanasia, or Ketamine/Xylazine for IP injection. The dosage of ketamine/xylazine used to induce anesthesia is typically 0.005 mL per gram of body weight. Euthanasia is induced by doubling this volume. (A 20 g mouse would receive 0.1 ml of drug for anesthesia, 0.2 ml for euthanasia).*

For Islet Isolation:

G-Solution STERILE FILTERED in 0.22 μm filter:

HBSS (Invitrogen #14065–056 diluted to 1X).

0.35 g NaHCO_3_/L.

1% Bovine Serum Albumin (BSA).

*This solution, as with any cell culture solution, must be sterile filtered. This method is performed using clean techniques, not sterile techniques; therefore, it is vital that the solutions are sterile prior to use.*

*Other protocols use protease inhibitors or varying concentrations of BSA to inactivate endogenous proteases, some as high as 10% BSA.*

*Prepare 100 mL G-solution per mouse.*

Collagenase Solution:

Solution (C): 1.4 mg/mL Collagenase-P (Roche #1129 002001) in G-Solution (prepare 5 mL/pancreas).

*Collagenase is an area where there are differences in protocols. We have chosen to maintain a constant concentration of 1.4 mg/mL and to vary the digestion time to suit different lot numbers. Studies use different collagenase types, but we ensure specific criteria are met by each lot prior to use. The digestion time is based on islet function and viability experiments before using a new enzyme lot experimentally.*

Gradient:

Histopaque 1100 Solution (1.100 g/mL):

100 mL Histopaque 1077 (SIGMA #10771).

120 mL Histopaque 1119 (SIGMA #11191).

*We prepare and store a stock solution at 4 °C. Only the amount of Histopaque required is brought to room temperature prior to its use. It is also important to note that the Histopaque solutions should be kept in the dark.*

Culture Media:

RPMI 1640 +L-Glutamine (Gibco #11875–093).

10% FBS (Gibco #16000–044).

Penicillin (100 U/mL)/Streptomycin (100 μg/mL) (Gibco #15140–122).

**Protocol**

1. Label all tubes, vials, and plates clearly and prepare all required volumes of solutions as well. Add 10 mL culture media to cell culture plates and place in 5% CO2 incubator set at 37 °C to warm.
2. Prepare 100 mL G-solution per mouse.
3. Place 25 mL of G-Solution into one set of the 50 mL conical tubes and set aside.
4. Prepare 1 mL of G-solution for each mouse in a 15 mL conical.
5. Place each 15 mL conical from Step 4 in ice.
  *To inhibit the collagenase digestion prior to incubation, place a 15 mL conical containing 1 mL of G-solution for each mouse on ice. The ice will maintain the collagenase-filled pancreata at a temperature which inhibits digestion. The ice also serves to maintain uniform conditions while isolating islets from multiple pancreata.*
6. Make up n+ 1 volume of collagenase solution (Solution C) and place in ice with 15 mL conical tubes.
  *The n+ 1 adds a layer of protection should more collagenase solution be required, especially for those just learning to cannulate the common bile duct.*
7. Euthanize animal with CO_2_ OR inject mouse IP with 0.01 mL/g body weight of Solution (B). The mouse is ready for exsanguination after no response to pinching its foot. NOTE: The investigator MUST refer to institution Animal Care and Use Committee guidelines and policies regarding proper procedures when handling and using animals in research.
  *Some researchers prefer to use ketamine/xylazine when collecting blood samples.*
8. (Optional) Exsanguinate the animal by heart perfusion using a 1 mL syringe with 25Ga needle or using preferred method.
  *Do not remove or disrupt the liver as this will destroy the common bile duct and remove the possibility of cannulation.*
9. Wet abdominal fur with 70% alcohol to reduce the chance of hair contamination in the IP cavity during subsequent steps.
10. Open abdomen with standard pattern scissors in a V-shape starting from the lower abdomen and extending to the lateral portions of the diaphragm in order to expose all organs in the peritoneal cavity.
11. Turn the animal so that the nose is closest to the surgeon and the tail points away from the surgeon.
  *This position allows for cannulation into the common bile duct from the rostral end closest to the liver at the junction of the cystic and hepatic ducts, leaving space to inject again closer to the duodenum if an error occurs.*
12. Secure the liver with 2"× 2" gauze.
  *Lay the liver flat and place an unfolded 2"× 2" piece of gauze under the liver closest to the surgeon. The gauze may be stretched across the xiphoid process and gently pressed down. This will cause the liver to become prominent and easier to gently flip over onto the gauze, exposing the junction of the gall bladder and common bile duct. Fold the remaining gauze over the liver to secure it during cannulation of the common bile duct.*
13. Using a Johns Hopkins Bulldog clamp (Roboz# RS-7441), clamp off the common bile duct (common bile duct) near the junction with the small intestine. Alternatively, tightly tied suture string can be used to tie off the common bile duct at the junction with the small intestine.
  *The pancreas contains a system of ducts that allows pancreatic enzymes to flow into the gut. The pancreatic duct merges with the inferior end of the common bile duct where the pancreatic enzymes enter the duodenum (see Fig.* *3**). This pancreatic duct provides the most readily available access to the endocrine pancreas. Clamp the common bile duct as close to the junction with the small intestine as possible so as not to occlude the pancreatic duct. Clamping the duodenum on either side of the junction with the common bile duct is also an option. Using a clamp allows access to the pancreatic duct while closing off access to the duodenum. Also attached to the common bile duct are several hepatic ducts. After becoming proficient with the perfusion technique, the major hepatic duct that leads to the caudal right lobe of the liver could also be clamped to ensure that minimal collagenase is delivered to the liver.*
14. Fill a 5 mL syringe with Solution (C).
15. Cannulate the common bile duct with a 27–30-gauge ½ in needle secured to a 5 mL syringe OR a 27–30-gauge butterfly needle. Cannulate the common bile duct at the junction of the cystic duct from the gall bladder and left hepatic duct from the liver (forms a Y-shape, see Fig. 3).
  *Needle size is often dictated by the size of the mouse. The average B6 mouse usually requires a 30-gauge needle while larger mice, such as a CD-1 will accommodate a 27-gauge needle. At the most superior portion of the common bile duct the junction of the cystic duct and hepatic duct forms a Y-shaped reference point in most mice. This location allows the surgeon the opportunity to cannulate the common bile duct with a 27-gauge butterfly needle OR a 27-gauge ½ in needle in 5 mL syringe. Tips for a successful cannulation of the duct: 1) use a new needle for each attempt because the needle may dull very easily; 2) place the needle at the Y-shaped junction and slide the needle into the duct moving in 1 mm increments (small-scale movement is the key to success); 3) if the duct is difficult to visualize, squeeze gently on the gall bladder which may cause the common bile duct to turn yellow as bile flows through.*
16. Inject 3-5 mL of Solution (C) into common bile duct.
  *A. An injection of 3-5 mL collagenase solution into the common bile duct should be sufficient to completely inflate the pancreas such that there is fluid in all regions of the pancreas from head to tail. The volume will be dictated by the size of the pancreas, with large mice requiring larger volumes. Ensuring that the collagenase inflates the pancreas in its entirety allows for the maximum yield of islets. The distribution of islets in the pancreas is not uniform; studies show a higher concentration of islets in the pancreatic tail, which makes the full inflation of the pancreas even more important. This step requires skill of the surgeon to obtain consistent results, and success is directly related to islet yield.*
  *B. Failed attempts to infuse the pancreas with collagenase through the hepatic duct eventually destroy the duct. An alternative method involves directly injecting collagenase into the numerous lobes of the pancreas. In this method, it is important to inject small amounts (about 1–2 drops) of collagenase into as many of the lobes of the pancreas as possible.*
17. Remove pancreas from mouse and place into labelled 15 mL conical from Step 5.
  *A. During the removal of the pancreas, it is important to avoid intestinal rupture to reduce the risk of contamination of the cell culture by intestinal bacteria. Removal begins at the point of attachment of the common bile duct to the duodenum. Using forceps or scissors snip the common bile duct and gently detach the pancreas from the intestines. Then, cut the common bile duct from the attachment to the liver. Continue to remove the pancreas from the greater curvature of the stomach and finally the spleen. Place the removed pancreas into the 15 mL conical that holds 1 mL of cold G-solution and replace the tube in the ice.*
  *B. After injecting the pancreas, place the pancreas that has been directly injected with collagenase into 1 mL of collagenase solution on ice, rather than G-solution, as in the preferred common bile duct-cannulation method above.*
18. Repeat the above steps for all mice, THEN proceed to the next step.
  *Follow the above procedure for the remaining animals placing one pancreas per tube on ice. Our lab recommends no more than six mice per isolation attempt.*
19. Incubate for 8*min. at 37 °C in 15 mL conical tube. *Incubation time varies among different lots of Collagenase (usually 7–10 min).
  *After collecting the pancreata into their respective tubes on ice, place the same tubes directly into a water bath at 37 °C to incubate for 7–10 min. The incubation time varies between lots of collagenase; therefore, it is important to test each lot prior to use in experimental conditions.*
20. Shake the 15 mL tube containing digested pancreas hard by hand for approximately 5 s to complete the separation of tissue (the result should be the consistency of pea soup and free of large pieces of pancreatic tissue if the pancreas was completely inflated).
  *This step results in the mechanical digestion of the pancreas and can be performed for all tubes simultaneously if the tubes are placed into a rack for incubation and covered while shaking. If fat tissue was incubated with the pancreas, it will remain undigested after shaking.*
21. Quickly fill to 15 mL with G-solution to dilute collagenase concentration and slow the digestive process.
22. Centrifuge 2 min at 1200 rpm (290xG).
23. Discard the supernatant by decanting into a waste container.
24. Wash with of 10 mL G-Solution, 2 min at 1200 rpm (290xG).
  *All wash steps, unless otherwise stated, involve resuspending the tissue pellet with a sterile 10-mL pipette using room temperature HBSS followed by room temperature centrifugation at 290xG for 2 min.*
25. Discard the supernatant by decanting into a waste container.
26. Add 10 mL of G solution and resuspend with a 10 mL pipette.
27. Filter each sample through a size 40 (420 μm) sieve (Bellco Glass, Inc., Cat# 1985–00040) into separate 50 mL conical tubes.
  *It is important to put each pancreas into its own conical so that the gradient can sustain the tissue load and properly separate the islets from the exocrine tissue. Overloading the gradient will lead to reduced islet yield and purity.*
28. Bring volume to 20 mL with G-solution.
29. Centrifuge at 1200 rpm (290xG) for 2 min to pellet resuspended tissue.
30. Decant supernatant and place the opening of the 50 mL conical onto a paper towel to remove as much liquid as possible.
  *Removing all the liquid ensures that the gradient density is not diluted when the pellet is resuspended in the gradient.*
31. Resuspend pellet in 15 mL Histopaque 1100 Solution with a 10 mL pipette.
  *Resuspending the tissue completely in the gradient ensures that islet density is not affected by attached exocrine tissue. Tissue filtered through the sieve should resuspend to a state that appears almost homogenous in the gradient.*
32. Centrifuge for 20 min at 1200 rpm (290xG). **NOTE: ISLETS are in the SUPERNATANT.**
  *After centrifugation, islets should be visible in the supernatant of the gradient as floating white specks. If there are fewer islets present than expected, resuspend the pellet produced during the previous step in order to inspect for islets. Also note that if more than one pancreas is added to the gradient, many islets will be found in the pellet. This should not be the case if the gradient contains only one mouse pancreas.*
33. Decant the supernatant into the 50 mL conical with 25 mL of G-solution set up in step 3**.**

**---NOTE: Collect all washes below to be able to look through them later.---**

34. Centrifuge at 1500 rpm (453xG) for 4 min and decant the supernatant. **NOTE: ISLETS are in the PELLET.**
  *Take care to pour the solution into the waste container in one motion to avoid having the solution wash back onto the pellet causing the islets to become dislodged and subsequently decanted.*
35. Add 10 mL of G-solution to the pellet and pipette up and down several times with a 10 mL pipette.
  *The final washes of the pellet are very important to ensuring that the islets are clear of the Histopaque solution and that the islets are well separated from one another prior to plating in culture dishes.*
36. Centrifuge at 1200 rpm (290xG) for 3 min.
37. Decant the supernatant and replace with 5 mL of pre-warmed culture medium using a new, sterile 5 mL serological pipette.
  *RPMI1640 with 10% FBS and 1% Pen/Strep*
38. Transfer to a sterile Petri dish under culture hood then place in incubator to recover for two hours before initial cleaning under a dissecting or light microscope.
  *Use a suspension (Petri) culture dish so that the islets do not stick as they would in a tissue-culture treated. Incubate or pick clean islets under dissecting or light microscope using low-retention pipette tips.*

## Appendix B: Bullet Point Protocol for Mouse Islet Isolation and Purification

Set up for dissection, scale, ruler, 2 forceps, 2 scissors, bulldog clamp, gauze, 1 mL and 5 mL syringes, and ice bucket.

Make sure water bath is on and set to 37 °C.

Label plates (3/mouse) and fill with RPMI (10 ml minimum). Place in 5% CO_2_ incubator at 37 °C.

Label one 15 ml and two 50 ml conical tubes per mouse.

Label 50 ml conical tube for Histopaque and add 15 ml of Histopaque per mouse.

Make 100 mL G-solution/mouse (90 mL HBSS + 10 mL BSA per mouse) in an appropriately sized beaker.

1 mL in each 15 mL conical and place on ice.

25 mL in each empty 50 mL conical (1/mouse).

5 mL (per mouse) + an extra 5 mL in the 50 mL beaker (n+ 1).

Place remaining G-solution with a 25 mL pipet near the centrifuge along with the 50 mL tube rack and waste bucket.

Make 5 mL collagenase solution/mouse (plus an extra 5 mL) add 7 mg/mouse of collagenase P to the 5 mL/mouse of G-solution in the 50 mL beaker ((n+ 1)*7).

Euthanize mouse per lab protocol.

Wet fur with alcohol, cut open abdomen up to forelegs, and turn animal with head closest to you. Pin rear feet if needed.

Pinch the end of one strip of gauze between thumb and index fingers and press on xyphoid process, scooping down and under the liver until it pops up. Fold the other end of the gauze over the liver to pin it down and expose the common bile duct.

Use Bulldog clamp to pinch white dot (ampulla of Vater) on small intestine, then lay clamp on the intestines on the left side.

Cannulate the common bile duct with collagenase solution. Longer bezel edge down, inject 4-5 mL or remove and pocket inject.

Remove pancreas and place in 15 mL conical.

Repeat steps 8–13 for all mice.

**Purification Protocol:**

*Pre-heat water bath*.

1. Digest @ 37° for 8 min.

2. Shake well to aid digestion.

3. Add G-solution to 15 mL mark.

Spin @1200 for 2 min.

4. Pour off supernatant, add 10 mL G-solution, resuspend.

Spin @1200 for 2 min.

5. Wet mesh to facilitate suspension flow.

6. Pour off supernatant, add 10 mL G-solution, resuspend/filter through metal mesh.

Add G-solution to 20 mL.

Spin @1200 for 2 min.

7. Pour off supernatant, tamp on towel.

Add 15 mL Histopaque 1100, resuspend.

Spin @1200 for **20** min.

8. **Keep supernatant** – pour into 50 mL tubes w/ 25 mL G-solution (discard pellet).

Spin @**1500** for **4** min.

9. Pour off supernatant, add 10 mL G-solution, resuspend.

Spin @**1200** for **3** min.

10. Pour off supernatant, resuspend with 10 mL warm RPMI and plate.

## Appendix C: Cell Death Fluorescent Imaging Protocol

1. Using a peristaltic pump, rinse the tubing by passing dH_2_O through an inline heater set at 37 °C into a diamond bath imaging chamber mounted to a stage adaptor on a BX51WIF fluorescence microscope with a 10x objective.
2. Next, in a 12-well plate, prepare combined Propidium Iodide (PI) and Annexin V (AnnV) dye using desired glucose solution (our lab uses a 3 mM glucose solution). PI, a cell exclusion dye, is used to detect generalized cell death. Apoptosis is measured using AnnV, which detect phospatidylserine when it is exposed to the outer leaflet of the plasma membrane during apoptosis.
  - Propidium iodide (MilliporeSigma, MA) dye solution is prepared by adding 7 ml of 3 mM stock PI solution to 700 ml of 3 mM glucose solution making a 30 mM final working concentration.
  - Annexing V fluorescent dye (Invitrogen, CA) is prepared by adding 35 ml of stock AnnV conjugate to 700 ml of 3 mM glucose solution.
  - Both PI and AnnV dye solution can be prepared in the same well as both dyes have different fluorescent wavelengths (700 ml glucose solution + 7 ml PI + 35 ml AnnV).
3. Place the plate in a 37 °C humidified atmosphere containing 5% CO_2_. Cover plate with aluminum foil to block out light, making certain sides are covered as well.
4. In the warmed 12-well plate, add 20–25 islets to the PI/AnnV prepared well. Replace plate into 37 °C humidified atmosphere containing 5% CO_2_ for 15 min.
5. While islets are incubating, change the peristaltic pump so that it is passing 3 mM glucose solution through the inline heater into the diamond bath imaging chamber rather than dH_2_O.
6. After 15 min incubation, place the islets into the diamond bath imaging chamber mounted on the microscope.
7. Stained islets are images using a Hamamatsu ORCA-Flash 4.0 digital camera mounted to s BX51WIF fluorescence microscope.
8. Excitation is provided using a xenon burner supplied to the image field through a light pipe and filter wheel with a Lambda 10–3 Optical Controller.
9. Regions of interest (ROI) are drawn around each islet and a brightfield (BF) image is taken. The white light source for the microscope is then turned off.
10. Using cellSens Dimension 1.13 imaging software, fluorescent images are taken with 488 nm excitation/ 525 nm emission for Annexin V (GFP, green fluorescence) and 535 nm excitation /620 nm emission for Propidium iodide (TRITC, red fluorescence).
  - Note islets with dense centers seen on the brightfield image denote hypoxic cores. Islets with hypoxic cores should be excluded from cell death analyses as they may represent false positives unrelated to treatment (see Fig. S1).
11. Repeat steps 4 through 10 for each treatment group. PI/AnnV fluorescent dye solution can be reused up to 4 incubation and measurements. Make fresh PI/AnnV solution for additional measurements.

When finished with all experiments, pass dH_2_O through tubing and chamber for 10 to 15 min to rinse out the system and shut down other equipment. Run air through tubing for an additional 10 min to dry tubing before shutting down peristaltic pump.

## Abbreviations

GSIS: Glucose-stimulated insulin secretion
AnnV: Annexin V
PI: Propidium iodide
FDA: Fluorescein diacetate
[Ca^2+^]_i_: Intracellular calcium
IL-1beta: Interleukin-1beta
IL-6: Interleukin-6
TNF-alpha: Tumor necrosis factor-alpha
