# Supplementary material for: A Practical Guide to Rodent Islet Isolation and Assessment Revisited
Source: Biol Proced Online. 2021 Mar 1;23:7. doi: 10.1186/s12575-021-00143-x (PMC7919091; doi:10.1186/s12575-021-00143-x)
Supplement: Supplementary file 1 — Additional file 1: Supplemental Fig. S1 Hypoxic cell death as an unwanted variable in PI/AnnV studies. Due to loss of vascular connection during isolation, islets can become severely hypoxic, resulting in necrosis, especially in the core of an islet. Identifying and removing such islets from experiments helps in acquiring accurate data. (A) Brightfield image of untreated (control) islets (top) and cytokine-treated islets (bottom; overnight treatment with 5 ng/mL IL-1b and 10 ng/mL TNF-a). The smaller islets in both images have no evidence of hypoxia while the larger islets have clearly defined dark centers. (B) All cytokine treated islets show a distinct ring of AnnV staining indicative of cytokine-induced apoptosis on the outermost layer of cells. Note the dark centers do not stain for AnnV. (C) PI staining showing both cytokine -associated and hypoxia-associated cell death. The only islet in the control showing PI staining is the large, dark centered islet while the cytokine treated islets show both cytokine-induced and hypoxia-associated cell death. The small cytokine treated islets on the left show several distinct punctate PI- stained nuclei typical of cytokine effects. The larger islets show varying degrees of punctate surface staining indicative of cytokine-induced cell death, but a large central mass of PI staining corresponding to the dark centers observed in the brightfield image. [file 12575_2021_143_MOESM1_ESM.docx]

**Figure S1.**


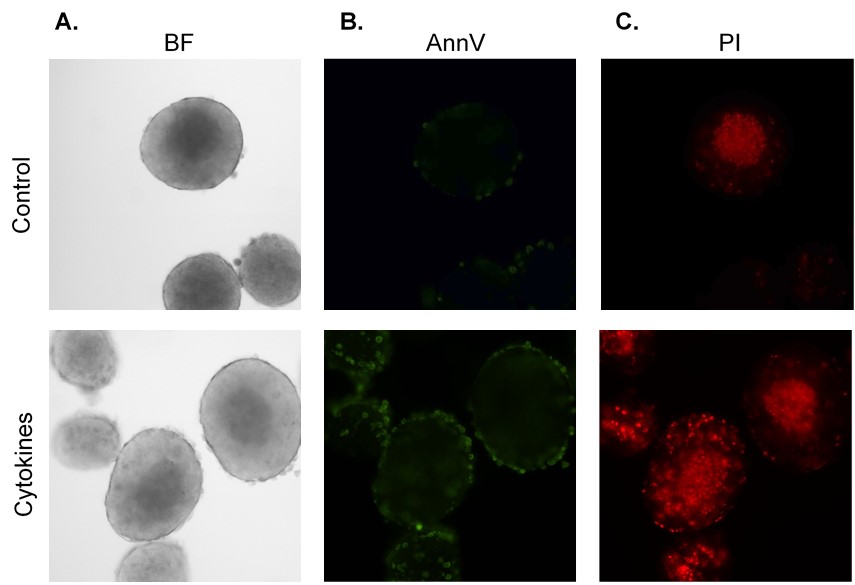


**Supplemental Figure S1**. Hypoxic cell death as an unwanted variable in PI/AnnV studies. Due to loss of vascular connection during isolation, islets can become severely hypoxic, resulting in necrosis, especially in the core of an islet. Identifying and removing such islets from experiments helps in acquiring accurate data. (A) Brightfield image of untreated (control) islets (top) and cytokine-treated islets (bottom; overnight treatment with 5 ng/mL IL-1b and 10 ng/mL TNF-a). The smaller islets in both images have no evidence of hypoxia while the larger islets have clearly defined dark centers. (B) All cytokine treated islets show a distinct ring of AnnV staining indicative of cytokine-induced apoptosis on the outermost layer of cells. Note the dark centers do not stain for AnnV. (C) PI staining showing both cytokine -associated and hypoxia-associated cell death. The only islet in the control showing PI staining is the large, dark centered islet while the cytokine treated islets show both cytokine-induced and hypoxia-associated cell death. The small cytokine treated islets on the left show several distinct punctate PI- stained nuclei typical of cytokine effects. The larger islets show varying degrees of punctate surface staining indicative of cytokine-induced cell death, but a large central mass of PI staining corresponding to the dark centers observed in the brightfield image.
